# Tetra­aqua­bis­[4-(pyrazin-2-ylsulfanylmeth­yl)benzoato]manganese(II) dihydrate

**DOI:** 10.1107/S160053681004506X

**Published:** 2010-11-06

**Authors:** Fu-An Li

**Affiliations:** a College of Chemistry and Chemical Engineering, Pingdingshan University, Pingdingshan 467000, Henan, People’s Republic of China

## Abstract

The title compound, [Mn(C_12_H_9_N_2_O_2_S)_2_(H_2_O)_4_]·2H_2_O, has been synthesized with a flexible asymmetrical bridging ligand, 4-(pyrazin-2-ylsulfanylmeth­yl)benzoic acid (Hpztmb). The Mn^II^ ion exhibits a centrosymmetric octa­hedral geometry involving two carboxyl­ate O atoms of two different pztmb ligands and four O atoms of four coordinated water mol­ecules. The packing shows a three-dimensional supra­molecular network *via* O—H⋯O and O—H⋯N hydrogen bonds and π–π stacking inter­actions [centroid–centroid distances = 3.884 (8) and 4.034 (8) Å] between the benzene ring of one pztmb anion and the pyrazine ring of an adjacent anion.

## Related literature

For background to the network topologies and applications of coordination polymers, see: Han *et al.* (2003 ▶, 2005 ▶, 2006 ▶); Zhao *et al.* (2002 ▶); Akutagawa & Nakamura (2000 ▶). For related syntheses and structures of a similar ligand (Hpmtmb), see: Han *et al.* (2006 ▶).
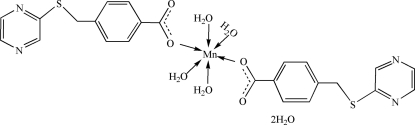


## Experimental

### 

#### Crystal data

[Mn(C_12_H_9_N_2_O_2_S)_2_(H_2_O)_4_]·2H_2_O
*M* *_r_* = 653.41
Monoclinic, 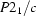

*a* = 16.587 (3) Å
*b* = 7.8928 (16) Å
*c* = 10.986 (2) Å
β = 94.38 (3)°
*V* = 1434.1 (5) Å^3^
*Z* = 2
Mo *K*α radiation
μ = 0.67 mm^−1^
*T* = 296 K
0.20 × 0.15 × 0.14 mm

#### Data collection

Bruker SMART APEXII CCD area-detector diffractometer
Absorption correction: multi-scan (*SADABS*; Bruker, 2005 ▶) *T*_min_ = 0.865, *T*_max_ = 0.925
17310 measured reflections
3425 independent reflections
3114 reflections with *I* > 2Σ(*I*)
*R*_int_ = 0.044

#### Refinement

*R*[*F* ^2^ > 2σ(*F* ^2^)] = 0.064
*wR*(*F* ^2^) = 0.229
*S* = 1.09
3425 reflections
188 parameters
H-atom parameters constrained
Δρ_max_ = 0.40 e Å^−3^
Δρ_min_ = −0.48 e Å^−3^

### 

Data collection: *APEX2* (Bruker, 2005 ▶); cell refinement: *SAINT* (Bruker, 2005 ▶); data reduction: *SAINT*; program(s) used to solve structure: *SHELXS97* (Sheldrick, 2008 ▶); program(s) used to refine structure: *SHELXL97* (Sheldrick, 2008 ▶); molecular graphics: *DIAMOND* (Brandenburg, 2010) ▶; software used to prepare material for publication: *SHELXTL* (Sheldrick, 2008 ▶).

## Supplementary Material

Crystal structure: contains datablocks I, global. DOI: 10.1107/S160053681004506X/pk2282sup1.cif

Structure factors: contains datablocks I. DOI: 10.1107/S160053681004506X/pk2282Isup2.hkl

Additional supplementary materials:  crystallographic information; 3D view; checkCIF report

## Figures and Tables

**Table 1:** Hydrogen-bond geometry (Å, °)

*D*—H⋯*A*	*D*—H	H⋯*A*	*D*⋯*A*	*D*—H⋯*A*
O1*W*—H1*WA*⋯O3*W*^i^	0.76	2.12	2.776 (4)	145
O1*W*—H1*WB*⋯O3*W*^ii^	0.97	1.78	2.716 (3)	162
O2*W*—H2*WA*⋯O2^iii^	0.85	2.06	2.878 (4)	162
O2*W*—H2*WB*⋯O2^iv^	0.85	1.95	2.752 (3)	157
O3*W*—H3*WA*⋯O2^v^	0.88	1.84	2.696 (4)	162
O3*W*—H3*WB*⋯N1^vi^	0.92	1.91	2.801 (4)	163

Symmetry codes: (i) 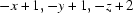; (ii) 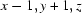; (iii) 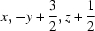; (iv) 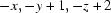; (v) 
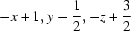
; (vi) 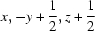.

**Fig. 1.**
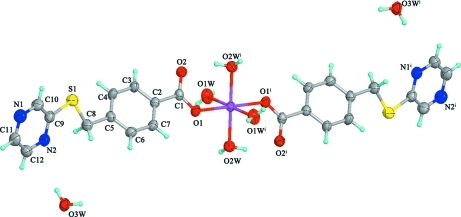
The molecular structure of the title compound, showing the atom-labelling scheme, with displacement ellipsoids drawn at the 50% probability level. Symmetry codes: (i) - x, 1 - y, 2 - z.

**Fig. 2.**
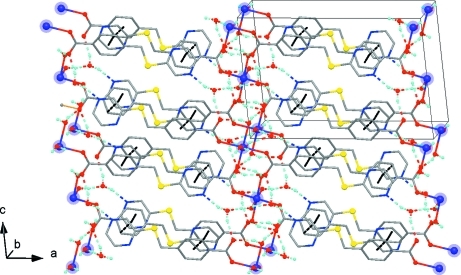
Three-dimensional supramolecular structure of the title compound. Hydrogen atoms have been omitted for clarity. Dashed lines indicate hydrogen-bonds and π···π interactions

## supplementary crystallographic information

### Comment

It is common knowledge that the coordination geometry of the metal ion and the shape and bonding mode of the ligand are generally the primary considerations in metal-mediated self-assembly reactions. Relatively small changes in the bridging ligand can give rise to large variation in the overall structure of the assembly. Recently, some coordination polymers containing long and flexible monoanionic ligands with hybrid pyridyl or pyrimidyl and benzoic carboxylate moieties have been reported (Han *et al.* 2005; Han *et al*. 2006). To better understand the influence of N-heterocyclic ring on the resultant structure, we have been working on the architectures of polymeric structures containing a novel long and flexible ligand 4-(2-pyrazinylthiomethyl)benzoic acid (Hpztmb). As part of our ongoing investigation, a new complex, [Mn(pztmb)_2_(H_2_O)_4_].2H_2_O, was prepared and its structure has been determined.

The title compound comprises one Mn^II^ ion, two pztmb anions, four coordinated water molecules and two solvent water molecules (Fig.1). The Mn^II^ ion has a centrosymmetric octahedral geometry coordinated by four O atoms from four coordinated water molecules and two carboxylate O1 atoms from two different pztmb anion ligands. In the crystal structure, in addition to hydrogen-bonds between the carboxylate O2 atoms and the solvent water molecules, hydrogen-bonds exist between coordinated and solvent water molecules and between coordinated water molecules and carboxylate O2 atoms (Table 1). Moreover, the solvent water molecules and the non-coordinated N1 atoms of pyrazine rings form O—H···N hydrogen-bonds (H3WB···N1^vi^ 1.91 Å). In addition, Two neighbouring pztmb anion ligands are parallel and arranged to enable π···π interaction (centroid-centroid distance of 4.034 (8) or 3.884 (8) Å) between the benzene ring of one pztmb anion and the pyrazine ring of an adjacent anion. Consequently, a variety of hydrogen-bonds and weak π···π interactions lead to a three-dimensional supramolecular network (Fig. 2).

### Experimental

A mixture of Mn(NO_3_)_2_.6H_2_O (28.5 mg, 0.1 mmol) with Hpztmb (50 mg, 0.2 mmol) in 10 ml of H_2_O was sealed in a stainless-steel reactor with a Teflon liner and heated at 110 K for 72 h. A quantity of colorless single crystals were obtained after the solution was cooled to room temperature at a rate of 10 K/h.

### Refinement

H atoms were positioned geometrically and refined using a riding model, with C—H = 0.93 Å, *U*_iso_(H) = 1.2Ueq(C) for aromatic H, and C—H = 0.97 Å, *U*_iso_(H) = 1.2Ueq(C) for CH_2_. Water H atoms were found in difference Fourier maps and initially included with a tight O—H restraint [0.85 Å]. In the final refinement, the positions of the water H atoms were fixed, with *U*_iso_(H) = 1.2Ueq(O).

### Crystal data

**Table:**

[Mn(C_12_H_9_N_2_O_2_S)_2_(H_2_O)_4_]·2H_2_O	*F*(000) = 677.8
*M**_r_* = 653.41	*D*_x_ = 1.513 Mg m^−^^3^
Monoclinic, *P*2_1_/*c*	Mo *K*α radiation, λ = 0.71073 Å
Hall symbol: -P 2ybc	Cell parameters from 786 reflections
*a* = 16.587 (3) Å	θ = 1.9–27.9°
*b* = 7.8928 (16) Å	µ = 0.67 mm^−^^1^
*c* = 10.986 (2) Å	*T* = 296 K
β = 94.38 (3)°	Prism, colourless
*V* = 1434.1 (5) Å^3^	0.2 × 0.15 × 0.14 mm
*Z* = 2	

### Data collection

**Table:**

Bruker SMART APEXII CCD area-detector diffractometer	3425 independent reflections
Radiation source: fine-focus sealed tube	3114 reflections with *I* > 2Σ(*I*)
graphite	*R*_int_ = 0.044
ω scans	θ_max_ = 27.9°, θ_min_ = 2.5°
Absorption correction: multi-scan (*SADABS*; Bruker, 2005)	*h* = −21→21
*T*_min_ = 0.865, *T*_max_ = 0.925	*k* = −10→10
17310 measured reflections	*l* = −14→14

### Refinement

**Table:**

Refinement on *F*^2^	Primary atom site location: structure-invariant direct methods
Least-squares matrix: full	Secondary atom site location: difference Fourier map
*R*[*F*^2^ > 2σ(*F*^2^)] = 0.064	Hydrogen site location: inferred from neighbouring sites
*wR*(*F*^2^) = 0.229	H-atom parameters constrained
*S* = 1.09	*w* = 1/[σ^2^(*F*_o_^2^) + (0.1555*P*)^2^] where *P* = (*F*_o_^2^ + 2*F*_c_^2^)/3
3425 reflections	(Δ/σ)_max_ = 0.004
188 parameters	Δρ_max_ = 0.40 e Å^−^^3^
0 restraints	Δρ_min_ = −0.47 e Å^−^^3^

### Special details

**Table:**

Geometry. All e.s.d.'s (except the e.s.d. in the dihedral angle between two l.s. planes) are estimated using the full covariance matrix. The cell e.s.d.'s are taken into account individually in the estimation of e.s.d.'s in distances, angles and torsion angles; correlations between e.s.d.'s in cell parameters are only used when they are defined by crystal symmetry. An approximate (isotropic) treatment of cell e.s.d.'s is used for estimating e.s.d.'s involving l.s. planes.
Refinement. Refinement of *F*^2^ against ALL reflections. The weighted *R*-factor *wR* and goodness of fit *S* are based on *F*^2^, conventional *R*-factors *R* are based on *F*, with *F* set to zero for negative *F*^2^. The threshold expression of *F*^2^ > σ(*F*^2^) is used only for calculating *R*-factors(gt) *etc*. and is not relevant to the choice of reflections for refinement. *R*-factors based on *F*^2^ are statistically about twice as large as those based on *F*, and *R*- factors based on ALL data will be even larger.

### Fractional atomic coordinates and isotropic or equivalent isotropic displacement parameters (Å^2^)

**Table:**

	*x*	*y*	*z*	*U*_iso_*/*U*_eq_	Occ. (<1)
Mn1	0.0000	0.5000	1.0000	0.0317 (3)	
O1	0.12369 (15)	0.5282 (3)	0.9481 (2)	0.0418 (6)	
O2	0.09688 (13)	0.6123 (3)	0.7547 (2)	0.0437 (6)	
O1W	−0.01101 (15)	0.7678 (3)	0.9589 (3)	0.0536 (7)	
H1WA	0.0092	0.8455	0.9892	0.064*	
H1WB	−0.0570	0.8395	0.9705	0.064*	
O2W	0.04302 (15)	0.5499 (4)	1.1920 (2)	0.0467 (6)	
H2WA	0.0503	0.6493	1.2206	0.056*	
H2WB	0.0076	0.4952	1.2278	0.056*	
O3W	0.88036 (15)	0.0223 (3)	0.9768 (2)	0.0457 (6)	
H3WA	0.8772	0.0607	0.9010	0.055*	
H3WB	0.8305	−0.0081	1.0013	0.055*	
N2	0.65704 (18)	0.7072 (4)	0.7820 (3)	0.0464 (7)	
N1	0.74665 (19)	0.5955 (5)	0.5930 (3)	0.0536 (8)	
C1	0.14473 (17)	0.5842 (4)	0.8483 (3)	0.0336 (6)	
C2	0.23309 (18)	0.6181 (4)	0.8372 (3)	0.0332 (7)	
C3	0.25917 (19)	0.7072 (4)	0.7380 (3)	0.0394 (7)	
H3	0.2217	0.7511	0.6789	0.047*	
C4	0.3410 (2)	0.7298 (5)	0.7279 (3)	0.0442 (8)	
H4	0.3580	0.7901	0.6618	0.053*	
C5	0.39827 (19)	0.6651 (4)	0.8138 (3)	0.0370 (7)	
C6	0.3719 (2)	0.5780 (5)	0.9120 (3)	0.0405 (7)	
H6	0.4095	0.5339	0.9707	0.049*	
C7	0.2902 (2)	0.5554 (5)	0.9244 (3)	0.0391 (7)	
H7	0.2734	0.4977	0.9918	0.047*	
C8	0.4882 (2)	0.6883 (5)	0.8047 (3)	0.0468 (8)	
H8A	0.5174	0.6071	0.8575	0.056*	
H8B	0.5037	0.8011	0.8327	0.056*	
C9	0.6222 (2)	0.6573 (4)	0.6757 (3)	0.0402 (7)	
C10	0.6666 (2)	0.6013 (5)	0.5810 (3)	0.0480 (9)	
H10	0.6396	0.5672	0.5079	0.058*	
C11	0.7822 (2)	0.6467 (5)	0.6995 (4)	0.0517 (9)	
H11	0.8383	0.6443	0.7114	0.062*	
C12	0.7382 (2)	0.7028 (5)	0.7917 (4)	0.0505 (9)	
H12	0.7655	0.7393	0.8641	0.061*	
S1	0.51581 (5)	0.66040 (14)	0.65082 (8)	0.0518 (4)	0.997 (4)

### Atomic displacement parameters (Å^2^)

**Table:**

	*U*^11^	*U*^22^	*U*^33^	*U*^12^	*U*^13^	*U*^23^
Mn1	0.0279 (4)	0.0358 (4)	0.0321 (4)	−0.0018 (2)	0.0060 (3)	−0.0009 (2)
O1	0.0301 (12)	0.0581 (15)	0.0384 (13)	−0.0014 (10)	0.0103 (10)	0.0082 (10)
O2	0.0294 (12)	0.0636 (16)	0.0384 (13)	0.0005 (10)	0.0038 (9)	0.0079 (10)
O1W	0.0436 (15)	0.0378 (13)	0.0800 (19)	0.0037 (10)	0.0090 (13)	0.0015 (13)
O2W	0.0422 (14)	0.0602 (15)	0.0383 (13)	−0.0095 (12)	0.0065 (10)	−0.0062 (11)
O3W	0.0342 (13)	0.0539 (15)	0.0504 (16)	−0.0006 (10)	0.0127 (11)	0.0088 (10)
N2	0.0366 (16)	0.0584 (19)	0.0453 (16)	−0.0020 (13)	0.0107 (12)	0.0020 (13)
N1	0.0378 (17)	0.070 (2)	0.055 (2)	0.0088 (15)	0.0144 (14)	0.0003 (16)
C1	0.0256 (14)	0.0343 (16)	0.0417 (17)	0.0015 (11)	0.0075 (12)	0.0022 (12)
C2	0.0283 (15)	0.0394 (16)	0.0325 (15)	0.0005 (12)	0.0061 (11)	−0.0008 (12)
C3	0.0289 (15)	0.0491 (19)	0.0405 (18)	−0.0021 (13)	0.0052 (12)	0.0116 (14)
C4	0.0317 (17)	0.058 (2)	0.0437 (19)	−0.0041 (14)	0.0091 (13)	0.0167 (15)
C5	0.0275 (15)	0.0464 (17)	0.0377 (16)	−0.0029 (12)	0.0056 (12)	−0.0005 (13)
C6	0.0357 (17)	0.051 (2)	0.0347 (16)	0.0029 (14)	0.0036 (13)	0.0043 (13)
C7	0.0365 (17)	0.0490 (18)	0.0326 (16)	−0.0031 (14)	0.0089 (13)	0.0042 (13)
C8	0.0324 (17)	0.069 (2)	0.0404 (19)	−0.0048 (15)	0.0079 (14)	−0.0002 (16)
C9	0.0343 (17)	0.0464 (18)	0.0411 (18)	0.0000 (13)	0.0101 (13)	0.0034 (13)
C10	0.0401 (19)	0.064 (2)	0.0406 (19)	0.0021 (16)	0.0089 (15)	−0.0023 (16)
C11	0.0275 (17)	0.071 (3)	0.057 (2)	0.0069 (15)	0.0097 (15)	0.0095 (18)
C12	0.0387 (19)	0.066 (2)	0.047 (2)	−0.0053 (16)	0.0020 (15)	0.0031 (17)
S1	0.0299 (5)	0.0853 (8)	0.0409 (6)	0.0005 (4)	0.0080 (4)	−0.0027 (4)

### Geometric parameters (Å, °)

**Table:**

Mn1—O1W	2.166 (3)	C2—C3	1.394 (4)
Mn1—O1W^i^	2.166 (3)	C3—C4	1.382 (4)
Mn1—O1	2.182 (2)	C3—H3	0.9300
Mn1—O1^i^	2.182 (2)	C4—C5	1.385 (5)
Mn1—O2W^i^	2.210 (2)	C4—H4	0.9300
Mn1—O2W	2.210 (2)	C5—C6	1.378 (4)
O1—C1	1.257 (4)	C5—C8	1.513 (4)
O2—C1	1.269 (4)	C6—C7	1.384 (5)
O1W—H1WA	0.7630	C6—H6	0.9300
O1W—H1WB	0.9660	C7—H7	0.9300
O2W—H2WA	0.8502	C8—S1	1.798 (4)
O2W—H2WB	0.8498	C8—H8A	0.9700
O3W—H3WA	0.8845	C8—H8B	0.9700
O3W—H3WB	0.9213	C9—C10	1.391 (5)
N2—C9	1.323 (5)	C9—S1	1.766 (3)
N2—C12	1.343 (4)	C10—H10	0.9300
N1—C10	1.324 (4)	C11—C12	1.367 (5)
N1—C11	1.333 (5)	C11—H11	0.9300
C1—C2	1.504 (4)	C12—H12	0.9300
C2—C7	1.385 (4)		
			
O1W—Mn1—O1W^i^	180.0	C2—C3—H3	120.2
O1W—Mn1—O1	84.97 (9)	C3—C4—C5	121.6 (3)
O1W^i^—Mn1—O1	95.03 (9)	C3—C4—H4	119.2
O1W—Mn1—O1^i^	95.03 (9)	C5—C4—H4	119.2
O1W^i^—Mn1—O1^i^	84.97 (9)	C6—C5—C4	118.4 (3)
O1—Mn1—O1^i^	180.00 (4)	C6—C5—C8	119.1 (3)
O1W—Mn1—O2W^i^	87.65 (11)	C4—C5—C8	122.5 (3)
O1W^i^—Mn1—O2W^i^	92.35 (11)	C5—C6—C7	120.9 (3)
O1—Mn1—O2W^i^	90.59 (9)	C5—C6—H6	119.5
O1^i^—Mn1—O2W^i^	89.41 (9)	C7—C6—H6	119.5
O1W—Mn1—O2W	92.35 (11)	C6—C7—C2	120.6 (3)
O1W^i^—Mn1—O2W	87.65 (11)	C6—C7—H7	119.7
O1—Mn1—O2W	89.41 (9)	C2—C7—H7	119.7
O1^i^—Mn1—O2W	90.59 (9)	C5—C8—S1	111.8 (2)
O2W^i^—Mn1—O2W	180.000 (1)	C5—C8—H8A	109.3
C1—O1—Mn1	126.4 (2)	S1—C8—H8A	109.3
Mn1—O1W—H1WA	131.8	C5—C8—H8B	109.3
Mn1—O1W—H1WB	126.8	S1—C8—H8B	109.3
H1WA—O1W—H1WB	78.3	H8A—C8—H8B	107.9
Mn1—O2W—H2WA	122.9	N2—C9—C10	122.3 (3)
Mn1—O2W—H2WB	99.6	N2—C9—S1	119.7 (3)
H2WA—O2W—H2WB	112.5	C10—C9—S1	118.0 (3)
H3WA—O3W—H3WB	112.0	N1—C10—C9	121.4 (3)
C9—N2—C12	115.5 (3)	N1—C10—H10	119.3
C10—N1—C11	116.7 (3)	C9—C10—H10	119.3
O1—C1—O2	124.8 (3)	N1—C11—C12	121.6 (3)
O1—C1—C2	118.1 (3)	N1—C11—H11	119.2
O2—C1—C2	117.2 (3)	C12—C11—H11	119.2
C7—C2—C3	119.0 (3)	N2—C12—C11	122.6 (4)
C7—C2—C1	120.0 (3)	N2—C12—H12	118.7
C3—C2—C1	121.0 (3)	C11—C12—H12	118.7
C4—C3—C2	119.6 (3)	C9—S1—C8	100.38 (16)
C4—C3—H3	120.2		
			
O1W—Mn1—O1—C1	−56.0 (3)	C5—C6—C7—C2	0.9 (5)
O1W^i^—Mn1—O1—C1	124.0 (3)	C3—C2—C7—C6	−1.2 (5)
O2W^i^—Mn1—O1—C1	31.6 (3)	C1—C2—C7—C6	176.6 (3)
O2W—Mn1—O1—C1	−148.4 (3)	C6—C5—C8—S1	139.1 (3)
Mn1—O1—C1—O2	−10.1 (5)	C4—C5—C8—S1	−41.8 (4)
Mn1—O1—C1—C2	171.00 (19)	C12—N2—C9—C10	1.1 (5)
O1—C1—C2—C7	13.5 (4)	C12—N2—C9—S1	−179.0 (3)
O2—C1—C2—C7	−165.5 (3)	C11—N1—C10—C9	−0.4 (6)
O1—C1—C2—C3	−168.7 (3)	N2—C9—C10—N1	−0.1 (6)
O2—C1—C2—C3	12.3 (5)	S1—C9—C10—N1	180.0 (3)
C7—C2—C3—C4	0.6 (5)	C10—N1—C11—C12	−0.1 (6)
C1—C2—C3—C4	−177.2 (3)	C9—N2—C12—C11	−1.6 (6)
C2—C3—C4—C5	0.4 (6)	N1—C11—C12—N2	1.2 (7)
C3—C4—C5—C6	−0.8 (6)	N2—C9—S1—C8	−13.5 (3)
C3—C4—C5—C8	−180.0 (3)	C10—C9—S1—C8	166.4 (3)
C4—C5—C6—C7	0.2 (5)	C5—C8—S1—C9	−170.0 (3)
C8—C5—C6—C7	179.4 (3)		

Symmetry codes: (i) −*x*, −*y*+1, −*z*+2.

### Hydrogen-bond geometry (Å, °)

**Table:**

*D*—H···*A*	*D*—H	H···*A*	*D*···*A*	*D*—H···*A*
O1W—H1WA···O3W^ii^	0.76	2.12	2.776 (4)	145
O1W—H1WB···O3W^iii^	0.97	1.78	2.716 (3)	162
O2W—H2WA···O2^iv^	0.85	2.06	2.878 (4)	162
O2W—H2WB···O2^i^	0.85	1.95	2.752 (3)	157
O3W—H3WA···O2^v^	0.88	1.84	2.696 (4)	162
O3W—H3WB···N1^vi^	0.92	1.91	2.801 (4)	163

Symmetry codes: (ii) −*x*+1, −*y*+1, −*z*+2; (iii) *x*−1, *y*+1, *z*; (iv) *x*, −*y*+3/2, *z*+1/2; (i) −*x*, −*y*+1, −*z*+2; (v) −*x*+1, *y*−1/2, −*z*+3/2; (vi) *x*, −*y*+1/2, *z*+1/2.
